# Immunohistochemical identification of complement peptide C5a receptor 1 (C5aR1) in non-neoplastic and neoplastic human tissues

**DOI:** 10.1371/journal.pone.0246939

**Published:** 2021-02-19

**Authors:** Benjamin Nürge, Alan Lennart Schulz, Daniel Kaemmerer, Jörg Sänger, Katja Evert, Stefan Schulz, Amelie Lupp

**Affiliations:** 1 Institute of Pharmacology and Toxicology, Jena University Hospital, Jena, Germany; 2 Department of General and Visceral Surgery, Zentralklinik Bad Berka, Bad Berka, Germany; 3 Laboratory of Pathology and Cytology Bad Berka, Bad Berka, Germany; 4 Department of Pathology, University of Regensburg, Regensburg, Germany; 5 Institute of Pathology, University Medicine of Greifswald, Greifswald, Germany; Western College of Veterinary Medicine, University of Saskatchewan, CANADA

## Abstract

The complement component C5a and its receptor C5aR1 are involved in the development of numerous inflammatory diseases. In addition to immune cells, C5aR1 is expressed in neoplastic cells of multiple tumour entities, where C5aR1 is associated with a higher proliferation rate, advanced tumour stage, and poor patient outcomes. The aim of the present study was to obtain a broad expression profile of C5aR1 in human non-neoplastic and neoplastic tissues, especially in tumour entities not investigated in this respect so far. For this purpose, we generated a novel polyclonal rabbit antibody, {5227}, against the carboxy-terminal tail of C5aR1. The antibody was initially characterised in Western blot analyses and immunocytochemistry using transfected human embryonic kidney (HEK) 293 cells. It was then applied to a large series of formalin-fixed, paraffin-embedded non-neoplastic and neoplastic human tissue samples. C5aR1 was strongly expressed by different types of immune cells in the majority of tissue samples investigated. C5aR1 was also present in alveolar macrophages, bronchial, gut, and bile duct epithelia, Kupffer cells, occasionally in hepatocytes, proximal renal tubule cells, placental syncytiotrophoblasts, and distinct stem cell populations of bone marrow. C5aR1 was also highly expressed in the vast majority of the 32 tumour entities investigated, where a hitherto unappreciated high prevalence of the receptor was detected in thyroid carcinomas, small-cell lung cancer, gastrointestinal stromal tumours, and endometrial carcinomas. In addition to confirming published findings, we found noticeable C5aR1 expression in many tumour entities for the first time. Here, it may serve as an interesting target for future therapies.

## Introduction

The complement system has important functions in innate immunity. These functions include opsonisation and phagocytosis, inflammation through recruitment of macrophages and neutrophils to sites of infection, and assembly of the membrane attack complex (MAC). Proteases in the complement system circulate as inactive precursors, which are sequentially activated by specific triggers, such as antigen-antibody complexes, foreign material, pathogens, damaged cells, or antigens, or by spontaneous hydrolysis of the complement component C3. Biochemical pathways regulating protease activation include the classical complement pathway, the alternative complement pathway, and the lectin pathway [1, 2]. One of the most important components of the complement cascade is C5a, which binds to the receptors C5aR1 (CD88) and C5aR2 (C5L2, GPR77). Activated C5aR1, a membrane-bound G-protein coupled receptor (GPCR), increases migration and adhesion of neutrophils and monocytes to vessel walls, resulting in the recruitment of immune cells to sites of infection or antigen-presenting cells to lymph nodes. Furthermore, activation of C5aR1 by C5a causes mast cell degranulation, cytokine release, and oxidative burst of immune cells [3–5]. The function of C5aR2, which is uncoupled from G proteins, remains controversial. As a decoy receptor for C5a, C5aR2 may prevent C5aR1-mediated immune cell activation. However, C5aR2 may also induce G-protein-independent signalling pathways, promoting anti- or pro-inflammatory actions [6–8].

The C5a/C5aR1 axis is involved in multiple inflammatory diseases, including allergic asthma, rheumatoid arthritis, inflammatory bowel disease, systemic lupus erythematosus, psoriasis, ischemia reperfusion injury, and sepsis [9, 10]. C5aR1 expression has also been described in a number of tumours. Here, it has been demonstrated to be associated with increased proliferation, metastasis, advanced tumour stage, and poor prognosis [11, 12]. Therefore, targeting C5a or C5aR1 may represent a promising therapeutic strategy for these tumour entities [11, 12].

Against this background, the objective of the present study was to evaluate C5aR1 expression by immunohistochemistry in a broad panel of non-neoplastic and neoplastic human tissue samples from routine pathology to obtain a broad expression profile of C5aR1 in these tissues and to provide a basis for more in-depth investigations with regard to diagnostic or therapeutic interventions also in tumour entities not investigated in this respect so far. Due to the extent of the study, we decided to generate and validate our own C5aR1 antibody which should be well-suited on one hand for immunohistochemical investigations on formalin-fixed, paraffin-embedded routine pathology samples but also for basic research in future projects on human cell lines and tissues using immunocytochemistry and Western blot analyses.

## Materials and methods

### Antibody

**Fig 1:**
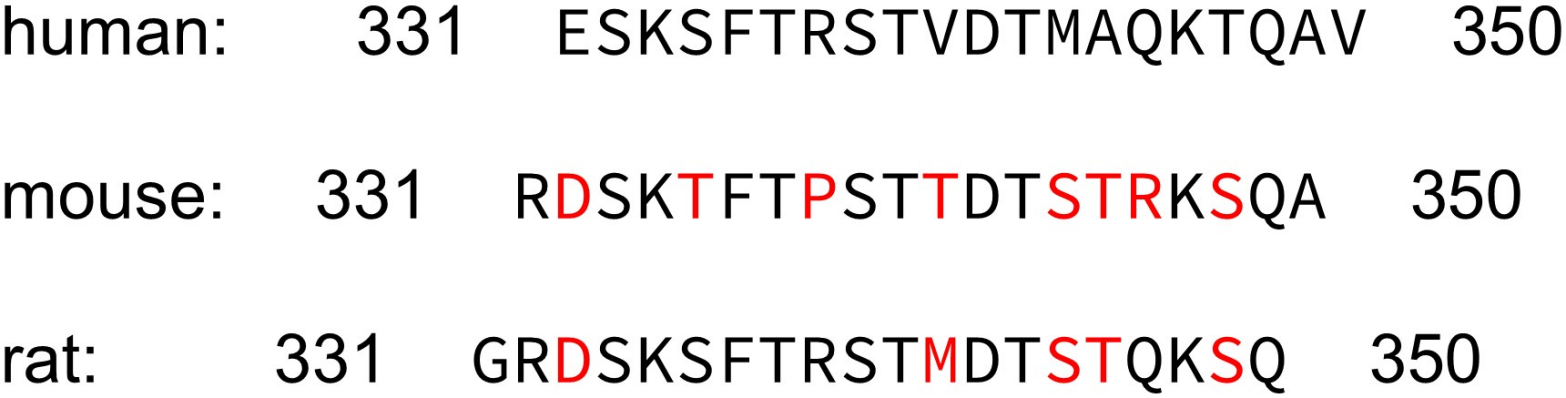
Carboxy-terminal sequence of human, mouse, and rat C5aR1. Differences in amino acid sequences of rat and mouse C5aR1 in comparison with the human receptor are marked in red.

A rabbit polyclonal antibody, {5227}, directed against the carboxy-terminal tail of human C5aR1 was generated in collaboration with and obtained from Thermo Fisher Scientific (Waltham, MA, USA). The identity of the peptide used for immunisation of rabbits was Cys-ESKSFTRSTVDTMAQKTQAV, which corresponds to residues 331–350 of human C5aR1. This sequence is unique to human C5aR1 and the antibody does not cross-react with rat or mouse C5aR1 due to dissimilarities in carboxyl-terminal amino acid sequences (Fig 1).

### Western blot analysis

Human embryonic 293 (HEK-293) cells were obtained from the German Collection of Microorganisms and Cell Cultures GmbH (ACC 305; Deutsche Sammlung von Mikroorganismen und Zellkulturen; DSMZ, Braunschweig, Germany) and were cultured up to the 10^th^ passage at most, to avoid genetic drift. Cell cultures were checked monthly by PCR for mycoplasma contamination. Cells were cultured at 37°C and 5% CO_2_ in Dulbecco’s modified Eagle’s medium (DMEM), supplemented with 10% fetal bovine serum, 2 mM L-glutamine and 100 U/ml penicillin/streptomycin. HEK293 cells were stably transfected with human C5aR1 or human C5aR2 using TurboFect (Thermo Fisher Scientific, Waltham, MA, USA). Stably transfected cells were selected in medium supplemented with 400 μg/ml geneticin (Thermo Fisher Scientific, Waltham, MA, USA). For Western blot analysis, cells were seeded onto poly-L-lysine-coated 60-mm dishes and grown to 80% confluence. Cells were lysed in detergent buffer [20 mM HEPES (pH 7.4), 150 mM NaCl, 5 mM EDTA, 1% Triton X-100, 10% glycerol, 0.1% SDS, 0.2 mM phenylmethylsulfonylfluoride, 10 mg/ml leupeptin, 1 mg/ml pepstatin A, 1 mg/ml aprotinin, and 10 mg/ml bacitracin]. C5aR1 and C5aR2 were enriched using wheat germ lectin agarose beads. Samples were subjected to 7.5% SDS-polyacrylamide gel electrophoresis and immunoblotted onto polyvinylidene fluoride (PVDF) membranes. Blots were incubated with rabbit polyclonal anti-C5aR1 antibody {5227} or rabbit polyclonal anti-C5aR2 antibody {5236} at a dilution of 1:500 followed by peroxidase-conjugated secondary goat anti-rabbit antibody at a 1:5,000 dilution (Santa Cruz Biotechnology, Dallas, TX, USA) and enhanced chemiluminescence detection (SuperSignal^®^ West Dura Extended Duration Substrate, Thermo Fisher Scientific, Waltham, MA, USA).

### Immunocytochemistry

HEK-293 cells stably transfected with human C5aR1 or human C5aR2 were grown on coverslips overnight. Cells were then fixed with 4% paraformaldehyde and 0.2% picric acid in phosphate buffer (pH 6.9) for 20 min at room temperature, washed with phosphate buffer, and incubated with rabbit polyclonal anti-C5aR1 antibody {5227} or rabbit polyclonal anti-C5aR2 antibody {5236} (1:500) at room temperature for 2 h, followed by an Alexa Fluor 488-conjugated secondary goat anti-rabbit antibody (Invitrogen, Karlsruhe, Germany; dilution: 1:5,000) overnight at 4°C. Samples were mounted and examined using a Zeiss LSM 510 META laser-scanning confocal microscope (Jena, Germany).

### Immunohistochemical evaluation of C5aR1 expression in non-neoplastic and neoplastic human tissues

#### Tissue specimens

C5aR1 expression was evaluated in 330 formalin-fixed and paraffin-embedded human tissue samples, consisting of 257 tumour specimens from 32 different tumour entities (n = 1–10 each; Table 1) and 73 tumour-free specimens from lung, heart, liver, gut, pancreas, kidney, spleen, tonsil, thymus, lymph node, placenta, and bone (n = 6–15 each). Many of the tumour specimens contained adjacent non-malignant tissues, which were also analysed. Staining patterns were compared to those in the non-malignant tissues surrounding tumours. Completely anonymised samples were obtained from the Department of Pathology at Ernst-Moritz-Arndt-University (Greifswald, Germany) and the Laboratory of Pathology and Cytology Bad Berka (Bad Berka, Germany). Permission was obtained from the local ethics committees (Ethikkommission an der Universitätsmedizin Greifswald; Ethikkommission der Landesärztekammer Thüringen) to access material from the pathology archives for the present study. For this type of study on anonymized human samples being archived for more than 10 years and which does not include clinical data, according to the local ethics committees formal consent was not required.

**Table 1:** Presence of C5aR1 in the tumour cells of different tumour entities.

Tumour type	C5aR1-positive tumours, n	Immunoreactive score (IRS)		
(total number of cases)		mean	min	max
Glioblastoma (8)	4	**3.44**	0	9
Meningioma (6)	5	**4.17**	0	6
Pituitary adenoma (10)	5	2.60	0	6
Thyroid carcinoma (37)	36	**5.76**	2	10
• papillary (10)	10	**5.60**	4	8
• follicular (10)	9	**4.50**	2	8
• medullary (9)	9	**5.44**	3	8
• anaplastic (8)	8	**7.50**	6	10
Lung cancer (30)	15	**3.15**	0	8
• Adenocarcinoma (10)	5	2.95	0	6
• Squamous cell carcinoma (10)	1	1.50	0	4.5
• Small-cell lung cancer (10)	9	**5.00**	2	8
Breast carcinoma (9)	2	1.22	0	4
Gastric adenocarcinoma (10)	8	**4.40**	0	6
Hepatocellular carcinoma (10)	10	**6.20**	4	10
Cholangiocellular carcinoma (9)	8	**6.77**	2	12
Pancreatic adenocarcinoma (10)	9	**4.55**	0	12
Pancreatic neuroendocrine tumour (10)	6	2.05	0	4.5
Colon carcinoma (9)	5	**3.06**	1.5	6
Gastrointestinal stromal tumour (9)	9	**8.44**	4	12
Pheochromocytoma (6)	0	0	0	0
Renal clear cell carcinoma (7)	7	**6.07**	4.5	8
Prostate adenocarcinoma (10)	1	1.60	0	6
Testicular cancer (10)	3	1.40	0	4
Ovarian cancer (9)	9	**7.89**	3	12
Endometrial cancer (10)	10	**5.60**	4	8
Cervical cancer (9)	8	**4.06**	2	6
Melanoma (5)	2	2.00	1	4
Lymphoma (10)	8	**3.70**	2	6
Sarcoma (14)	3	1.36	0	6
• Leiomyosarcoma (4)	0	0.50	0	2
• Rhabdomyosarcoma (3)	2	**3.00**	0	6
• Pleomorphic sarcoma (2)	0	1.00	0	2
• Liposarcoma (4)	0	0	0	0
• Angiosarcoma (1)	1	**6.00**	6	6

#### Immunohistochemistry

From the paraffin blocks, 4-μm sections were prepared and floated onto positively charged slides. Immunostaining was performed by an indirect peroxidase-labelling method as previously described [13]. Briefly, sections were dewaxed, microwaved in 10 mM citric acid (pH 6.0) for 16 min at 600 W, and incubated with {5227} (dilution: 1:500) overnight at 4°C. Detection of the primary antibody was performed using biotinylated goat anti-rabbit IgG (Vector ABC “Elite” kit; Vector Laboratories, Burlingame, CA, USA) followed by incubation with peroxidase-conjugated avidin (Vector ABC “Elite” kit; Vector Laboratories, Burlingame, CA, USA). Binding of the primary antibody was visualised using 3-amino-9-ethylcarbazole in acetate buffer (BioGenex, San Ramon, CA, USA). Sections were rinsed, counterstained with Mayer’s haematoxylin, and mounted in Vectamount mounting medium (Vector Laboratories, Burlingame, CA, USA). For immunohistochemical controls, {5227} was either omitted or adsorbed for 2 h at room temperature with 10 μg/ml of the peptide used for immunisations.

C5aR1 tumour staining was scored using the semi-quantitative Immunoreactivity Score (IRS) according to Remmele and Stegner [14]. The percentage of positive tumour cells assessed in five gradations (no positive cells (0), <10% positive cells (1), 10–50% positive cells (2), 51–80% positive cells (3), and >80% positive cells (4)) was multiplied by the staining intensity quantified in four gradations (no staining (0), mild staining (1), moderate staining (2), and strong staining (3)). Thus, IRS values ranging from 0 to 12 were obtained. Two independent, blinded investigators (BN, AL) evaluated all stained slides. Final decisions on discrepant scores were achieved by consensus.

## Results

### Characterisation of rabbit anti-human C5aR1 antibody {5227}

**Fig 2:**
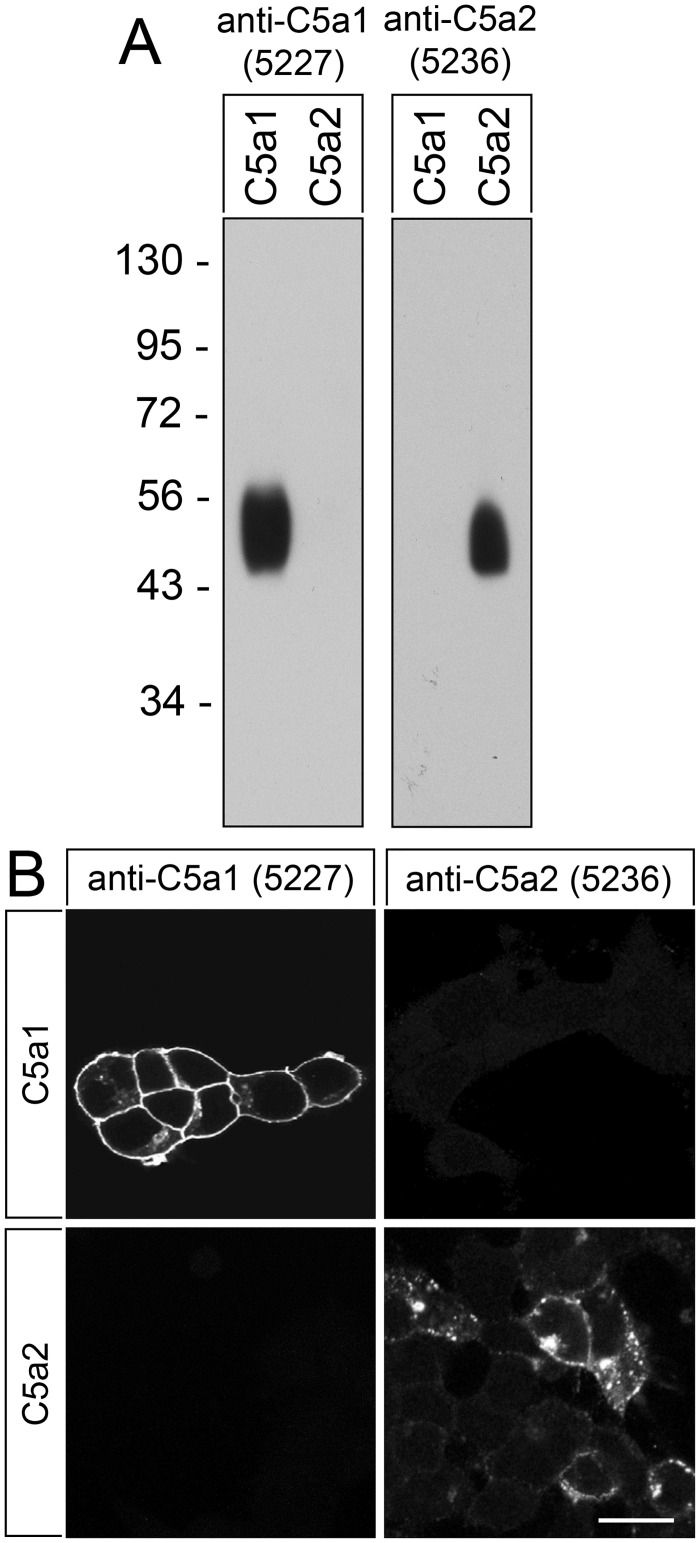
Analysis of the specificity of anti-human rabbit polyclonal antibody {5227}. (A) Western blot analysis of whole cell preparations from stably C5aR1- or C5aR2-transfected HEK-293 cells. Receptors were enriched using wheat germ lectin agarose beads. Samples were separated on 7.5% SDS-polyacrylamide gels and blotted onto PVDF membranes. Membranes were then incubated with anti-human C5aR1 antibody {5227} or anti-human C5aR2 antibody {5236}, and blots were developed using enhanced chemiluminescence. Ordinate, migration of protein molecular weight markers (kDa). Note that {5227} and {5236} selectively detected only the respective targeted receptor and did not cross-react with the other receptor or other membrane proteins. (B) Immunocytochemistry of stably C5aR1- or C5aR2-transfected HEK-293 cells. Cells were fixed and immunofluorescently stained with anti-human C5aR1 antibody {5227} or anti-human C5aR2 antibody {5236}. Again, {5227} and {5236} selectively detected only the targeted receptor and did not cross-react with the other receptor. Note also, that prominent immunofluorescence for C5aR1 was localised at the plasma membrane. Representative results from one of three independent experiments are shown. Scale bar: 20 μm.

The specificity of anti-human C5aR1 antibody {5227} was first tested by Western blot analysis of transfected HEK-293 cells. The anti-C5aR1 antibody {5227} detected a broad band migrating at 45–58 kDa, corresponding to the expected molecular weight range of the variably glycosylated receptor, in cells stably expressing C5aR1 but not in cells expressing C5aR2 (Fig 2A, left panel). Conversely, the rabbit polyclonal anti-human C5aR2 antibody {5236} detected a broad band migrating at 45–56 kDa in cells expressing C5aR2 but not in cells expressing C5aR1 (Fig 2A, right panel). The anti-C5aR1 antibody {5227} was further characterised using immunofluorescent staining of transfected cells. The anti-C5aR1 antibody {5227} demonstrated prominent immunofluorescence localisation at the plasma membrane in C5aR1-expressing cells but not in C5aR2-expressing cells (Fig 2B). On the other hand, the anti-C5aR2 antibody {5236} revealed punctate cytoplasmic immunofluorescence only in C5aR2-expressing and not in C5aR1-expressing cells (Fig 2B). This punctate cytoplasmic immunofluorescence most likely reflects localization of C5aR2 in endosomal vesicles, which would be in line with the function of C5aR2 as decoy receptor.

### Immunohistochemical detection of C5aR1 expression in non-neoplastic and neoplastic human tissues

The rabbit polyclonal anti-C5aR1 antibody {5227} was then subjected to immunohistochemical staining of a large panel of non-neoplastic and neoplastic human tissues. In all positive cases, the immunostaining was confined to the plasma membrane and to the cytoplasm of the cells. A set of both non-neoplastic and neoplastic tissues showing positive staining for C5aR1 was also incubated with {5227} pre-adsorbed with the immunising peptide, which, in all cases, led to complete abolishment of the immunosignal (see insets in Figs 4B, 5A, 5C-5E, 6A, 6B, 6D, and 6E).

**Fig 3:**
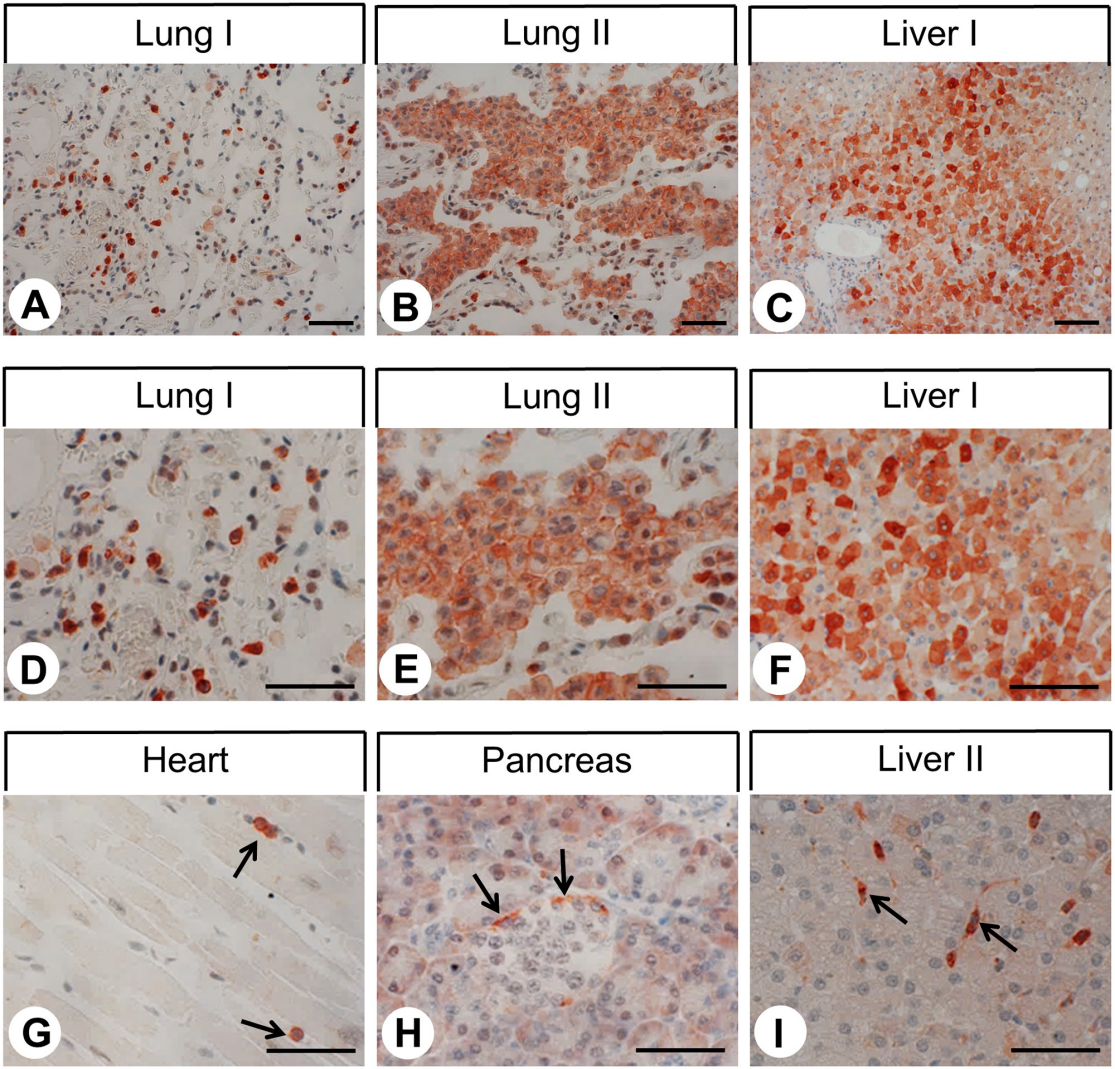
Immunohistochemical detection of C5aR1 localisation in non-neoplastic human tissues (I). Immunohistochemical staining (red-brown colour), counterstaining with haematoxylin. Scale bar: 100 μm (C, F), 50 μm (A, B, D, E, G-I). Arrows in (G): positive immune cells in the heart; arrows in (H): positive cells at the margin of the pancreatic islet; arrows in (I): positive Kupffer cells in the liver. (D-F) enlarged sections of (A-C).

**Fig 4:**
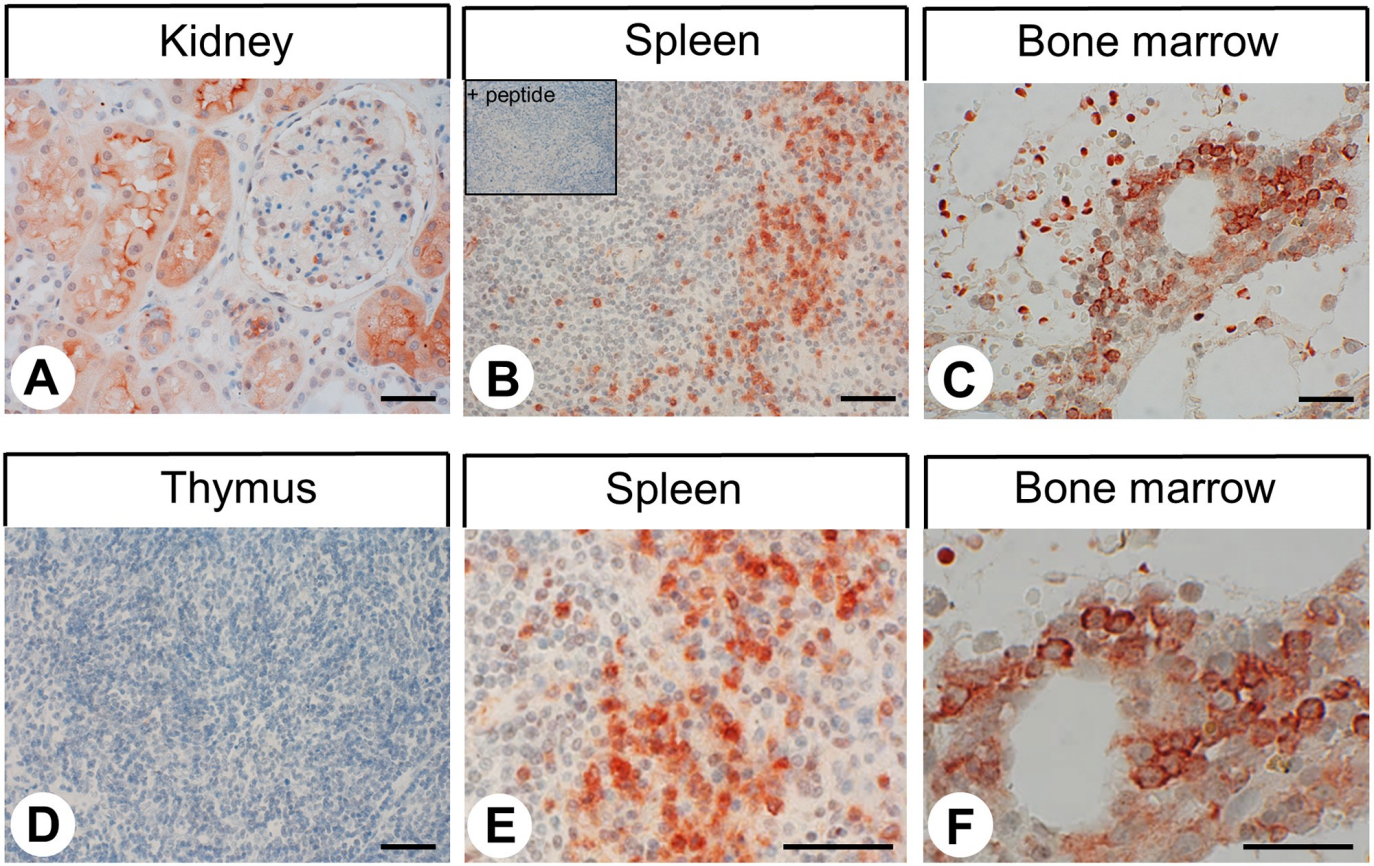
Immunohistochemical detection of C5aR1 localisation in non-neoplastic human tissues (II). Immunohistochemical staining (red-brown colour), counterstaining with haematoxylin. Scale bar: 50 μm (A, D, C, E), 30 μm (D, F). Inset in (B): for adsorption control, the anti-C5aR1 antibody {5227} was incubated with 10 μg/ml of the peptide used for immunisations. (E and F) enlarged sections of (B and C).

Various types of strongly C5aR1-positive immune cells were detected in nearly all non-neoplastic and neoplastic tissue samples. Based on localisation and morphology, these cells most likely represent monocytes, macrophages, granulocytes, dendritic cells, and mast cells. In addition to other immune cells, alveolar macrophages in the lung displayed strong C5aR1 expression (Fig 3A, 3B, 3D and 3E). C5aR1 staining was also noted in the epithelia of the airways. The heart was mostly negative except for immune cells within the lumen of vessels (Fig 3G, arrows). Whereas in the exocrine pancreas no or only faint staining was observed, a few cells at the outer edge of the pancreatic islets showed strong C5aR1 expression (Fig 3H, arrows). Kupffer cells in the liver showed strong C5aR1 immunostaining (Fig 3I, arrows). Bile duct epithelia were also C5aR1-positive. Seven of the 15 liver tissue samples showed C5aR1 expression in the hepatocytes, primarily those in periportal areas (Fig 3C and 3F). In the other eight of the liver tissue samples, however, hepatocytes were largely negative (Fig 3I). In the kidneys, C5aR1 expression was confined to the epithelial cells of the proximal tubules (Fig 4A). C5aR1 positivity was also observed in the epithelia of the duodenum, jejunum, ileum, and colon and in syncytiotrophoblasts of placenta (not shown). C5aR1 was detected in distinct cell populations of the red pulp of the spleen, most probably representing granulocytes and macrophages (Fig 4B and 4E), whereas the thymus (Fig 4D) and the white pulp of the spleen were largely negative. Similarly, the tonsils and lymph nodes were largely negative except for dendritic cells in the germ centres of secondary follicles and lymphocytes and macrophages in reactive lymph follicles (e.g., in the vicinity of tumour cells of tumour-bearing lymph nodes) (Fig 6C, arrowheads). Finally, positive immunostaining was detected in distinct cell populations within the bone marrow (Fig 4C and 4F), whereas osteoblasts, osteocytes, and osteoclasts in bone tissue were negative.

**Fig 5:**
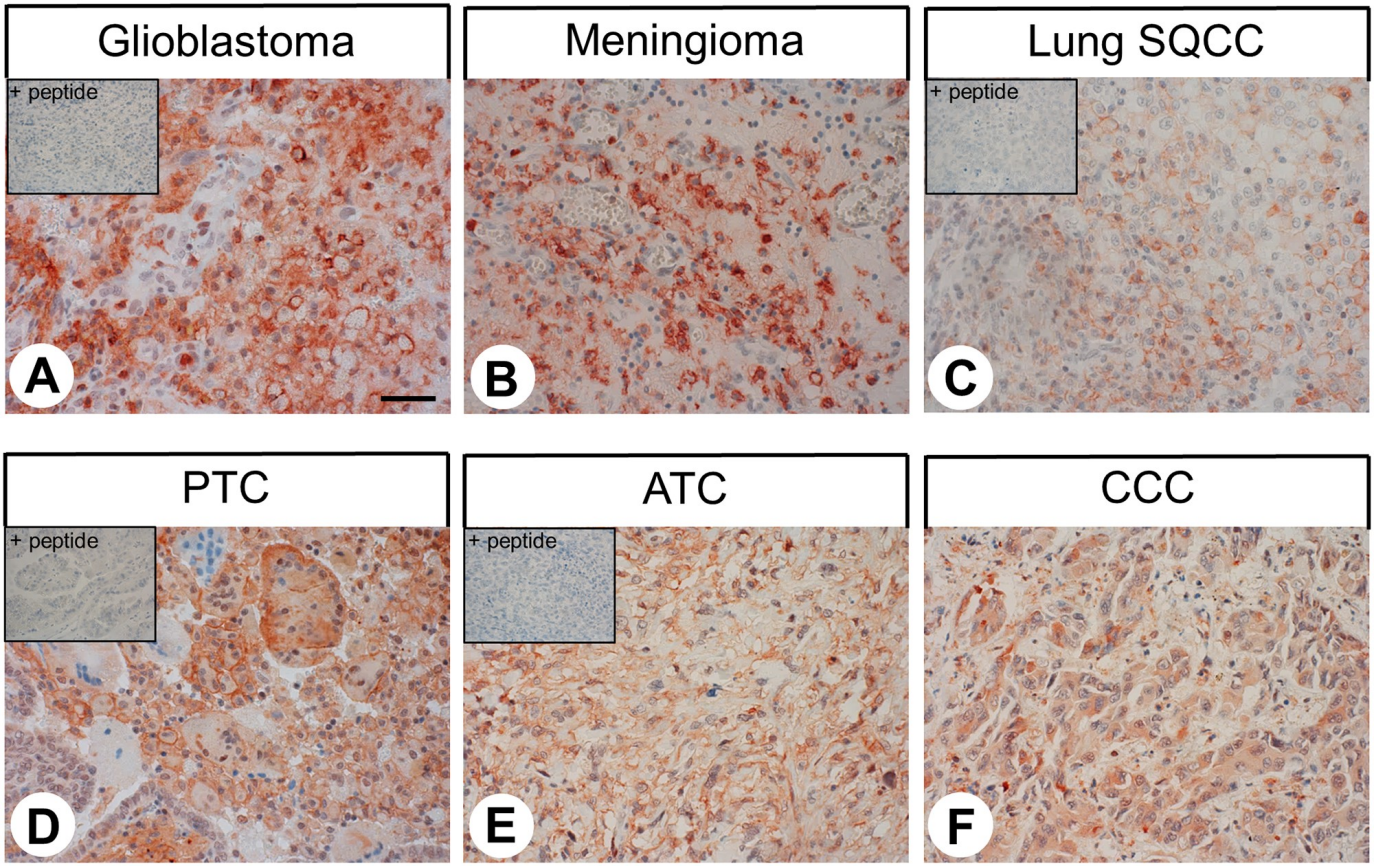
Immunohistochemical detection of C5aR1 localisation in human tumour entities (I). Immunohistochemical staining (red-brown colour), counterstaining with haematoxylin. Scale bar: 50 μm (A-F). Insets in (A, C, D, E): for adsorption control, the anti-C5aR1 antibody {5227} was incubated with 10 μg/ml of the peptide used for immunisations. Lung SQCC: squamous cell carcinoma of the lung; PTC: papillary thyroid carcinoma; ATC: anaplastic thyroid carcinoma; CCC: cholangiocellular carcinoma.

**Fig 6:**
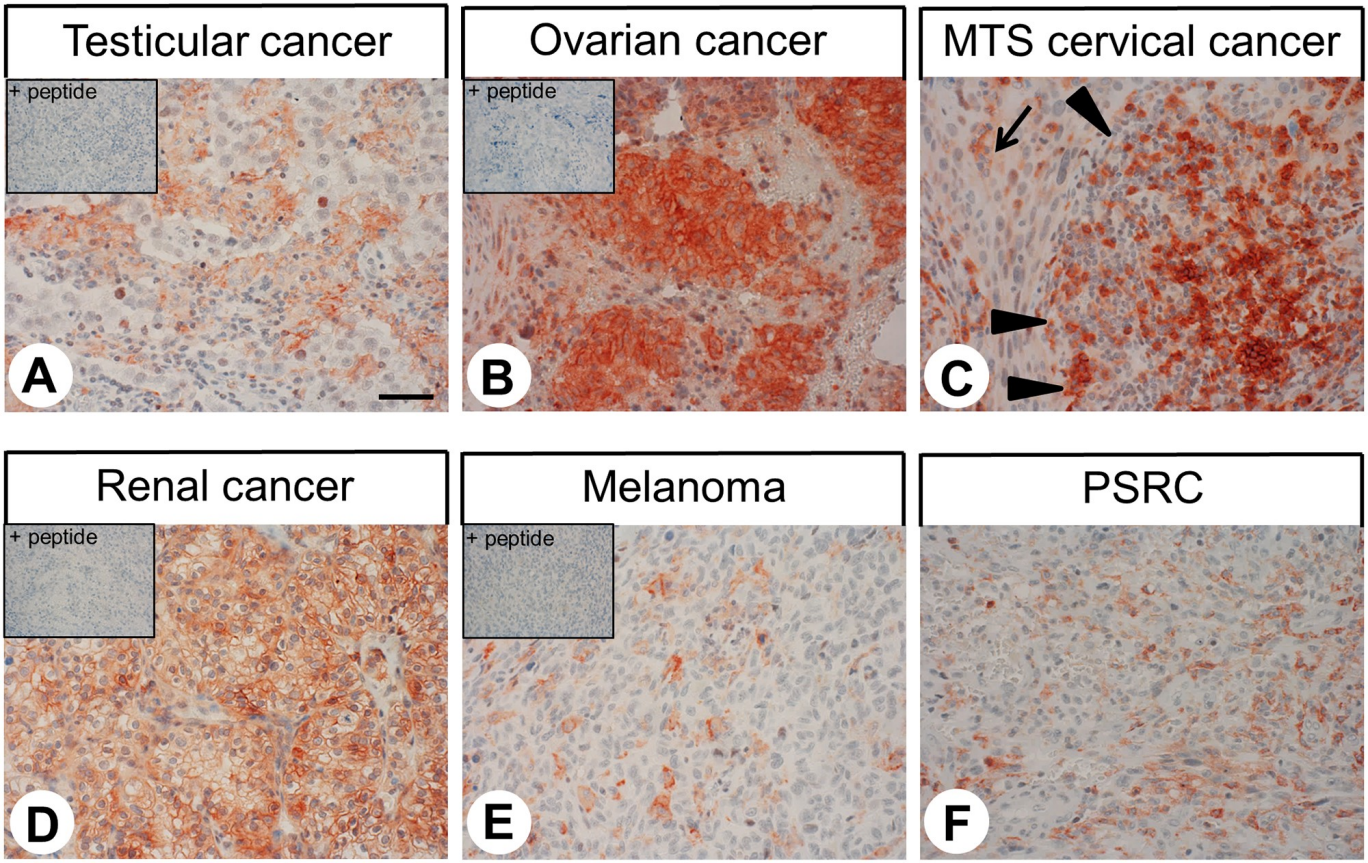
Immunohistochemical detection of C5aR1 localisation in human tumour entities (II). Immunohistochemical staining (red-brown colour), counterstaining with haematoxylin. Scale bar: 50 μm (A-F). Insets in (A, B, D, E): for adsorption control, the anti-C5aR1 antibody {5227} was incubated with 10 μg/ml of the peptide used for immunisations. Arrow in (C): tumour cells (lymph node metastasis of a cervical carcinoma); arrowheads in (C): lymph follicle. MTS: lymph node metastasis; PSRC: pleomorphic sarcoma.

The patterns of C5aR1 distribution in human tumour samples are summarised in Table 1. Representative examples of immunostaining are shown in Figs 5 and 6. Also here, both membranous and cytoplasmic staining of cells was observed. Notably, C5aR1 expression in the different tumour entities displayed substantial inter-individual variability. Overall, significant C5aR1 expression was observed in the majority of the 32 tumour entities investigated. Only a few tumour entities, such as pituitary adenomas, adenocarcinomas and squamous cell carcinomas of the lung, breast carcinomas, pancreatic neuroendocrine tumours, pheochromocytomas, prostate adenocarcinomas, testicular carcinomas, melanomas, and sarcomas were largely negative. By contrast, pronounced C5aR1 expression in a higher number of tumour samples was observed in all types of thyroid carcinomas, small-cell lung cancer, hepatocellular and cholangiocellular carcinomas, pancreatic adenocarcinomas, gastrointestinal stromal tumours, renal clear cell carcinomas and ovarian and endometrial cancer.

## Discussion

### Characterisation of rabbit anti-human C5aR1 antibody {5227}

The primary objective of the present study was to obtain a broad expression profile of C5aR1 in human non-neoplastic and neoplastic tissues and to provide a basis for future more in-depth investigations with regard to diagnostic or therapeutic interventions also in tumour entities not investigated in this respect so far. Due to the extent of the study, we decided to generate and thoroughly characterise our own C5aR1 antibody which should be well-suited on one hand for immunohistochemical investigations on formalin-fixed, paraffin-embedded routine pathology samples but also for basic research on human cell lines and tissues using immunocytochemistry and Western blot analyses.

We were able to demonstrate that our novel anti-C5aR1 antibody {5227} specifically detects its target and does not cross-react with other proteins. First, in Western blot analyses, the anti-C5aR1 antibody selectively detected its cognate receptor at the expected molecular weight of the glycosylated receptor in crude extracts from C5aR1-transfected HEK-293 [15, 16] but not in extracts from C5aR2-transfected cells. Second, the antibody revealed membranous staining of C5aR1-transfected HEK-293 cells, whereas no immunostaining was observed in C5aR2-transfected cells. Third, the novel anti-C5aR1 antibody {5227} showed highly efficient membranous and cytoplasmic staining of distinct cell populations known to express C5aR1 within formalin-fixed, paraffin-embedded human tissue samples. This staining was completely abolished after pre-adsorption of the antibody with its immunising peptide.

### Immunohistochemical detection of C5aR1 expression in non-neoplastic human tissues

C5aR1 is known to be expressed in distinct immune cell populations, such as monocytes, macrophages, monocyte-derived dendritic cells, eosinophilic and neutrophilic granulocytes, and mast cells [17–20]. The results of the current study agree with these findings. Based on our results showing negative staining in lymph nodes, the white pulp of the spleen, the tonsils, and thymus glands, resting B and T lymphocytes do not express C5aR1, which also corresponds well with previous reports [17–20]. As evident from the cervical cancer lymph node metastasis depicted in Fig 6C, C5aR1 may, however, be present in memory B cells and/or plasma cells [21].

In addition to the above-mentioned immune cells [20, 22–25], alveolar macrophages and bronchial epithelial cells have been shown to be C5aR1-positive [16, 22], which is consistent with our results. An increased concentration of C5a and an elevated amount of neutrophilic and eosinophilic granulocytes has been detected in the bronchoalveolar lavage fluid of asthma patients [26]. Furthermore, anti-C5aR1 treatment reduced the amount of neutrophilic and eosinophilic granulocytes in the bronchoalveolar lavage fluid of a mouse model of allergic asthma [27]. Therefore, C5a and C5aR1 may be interesting targets for treating asthma [9, 28].

In seven of the 15 liver samples, pronounced C5aR1 expression was detected in a distinct hepatocyte subpopulation in the periportal areas of the liver lobules. By contrast, the remaining eight liver samples were largely negative, except for infiltrating immune cells, Kupffer cells, and bile duct epithelia. These results agree with literature data showing that C5aR1 is not expressed in normal rat or human hepatocytes [29–32] in contrast to Kupffer cells [31, 32] and bile duct epithelia [11]. Under inflammatory conditions, however, C5aR1 expression can be distinctly induced in hepatocytes of mice, rats, and humans [22, 31, 32]. Upregulation of C5aR1 expression has also been shown to occur in the regenerating liver of rats after partial hepatectomy [33]. It is therefore likely that inflammatory and/or regenerative processes were present in the positively stained samples of the current study.

Pancreatic tissues showed only a few C5aR1-positive cells, which were located on the outer edge of the islets. These cells may represent glucagon-, somatostatin-, or pancreatic polypeptide-producing cells. The insulin-producing beta cells, however, which represent the majority of islet cells, were negative. Similar, low C5aR1 mRNA expression in human and mouse pancreatic islets has been described [34]. The authors of that study also demonstrated that treatment of islet cells *in vitro* with C5a or a C5aR1 agonist increases intracellular calcium and ATP concentrations, potentiates glucose-induced insulin secretion, and protects cells from apoptosis, whereas treatment with a C5aR1 antagonist caused a decrease in insulin secretion. Therefore the authors suggested that C5aR1 is expressed on beta cells, and mild inflammation may have a positive impact on glucose homeostasis via a direct effect on these cells [34]. In an animal model of diet-induced obesity, however, no difference in pancreatic islet morphology and beta cell mass was observed between wild-type and C5aR1-knockout animals, although C5aR1-deficient mice exhibited improved insulin sensitivity and fewer macrophages, particularly pro-inflammatory M1 macrophages, in the adipose tissue [35]. Thus, further investigation about the nature of C5aR1-expressing islet cells is needed.

C5aR1 expression was also observed in proximal renal tubule cells, which is consistent with previous reports [11, 29, 30, 36].

Results regarding C5aR1 expression in the non-neoplastic gut are controversial. Similar to our results, one report showed C5aR1 in epithelia of the small bowel and colon of mouse and pavian [22]. However, other studies did not find C5aR1 expression in human samples [11, 37]. Further investigations are necessary to clarify these discrepancies. Distinct C5aR1 expression was also observed in the syncytiotrophoblasts of the placenta, which, again, corresponds well with recent findings in the literature [38]. Here, the C5a –C5aR1 axis has been related to different pathologies, such as placental dysfunction, pre-eclampsia, intrauterine growth restriction, and spontaneous abortion [38–40]. In bone marrow, C5aR1 expression has been demonstrated on mesenchymal stem cells and myeloid but not lymphatic hematopoietic precursor cells [17, 41], which is consistent with our findings. Here, the C5a –C5aR1 axis seems to be involved in the mobilization of stem cells into the blood and in the recruitment of these cells to damaged tissues [41, 42].

### Immunohistochemical detection of C5aR1 expression in neoplastic human tissues

In the present study, 32 different tumour entities were evaluated for C5aR1 expression. In the majority of tumour types, a high percentage of cases showed significant C5aR1 expression. For many entities, literature data could be confirmed, e.g., for adenocarcinomas and squamous cell carcinomas of the lung [43–45], breast cancer [11, 46], gastric adenocarcinomas [11, 47–49], hepatocellular and cholangiocellular carcinomas [11, 50], pancreatic adenocarcinomas [11], colon carcinomas [11], renal clear cell cancer [11, 51], prostate adenocarcinomas [11], cervical cancer [52], and ovarian cancer [53]. In some studies, the presence of the C5aR1 was associated with a higher proliferation rate, metastatic disease, advanced tumour stage, and poor patient outcomes [43, 44, 46–51, 53]. Furthermore, in animal models of lung cancer, gastric cancer, or ovarian cancer, anti-C5a treatment or C5aR1 knockdown, silencing or inhibition decreased proliferation, tumour vascularisation, tumour growth, and metastatic burden [44, 49, 53, 54]. Thus, C5aR1 may represent an interesting therapeutic target for many tumour entities [12].

Among the 32 tumour entities investigated in the present study, 19 were evaluated for C5aR1 expression for the first time. These tumour types were glioblastomas, meningiomas, pituitary adenomas, papillary, follicular, medullary and anaplastic thyroid carcinomas, small cell lung cancer, gastrointestinal stromal tumours, pheochromocytomas, testicular cancer, endometrial carcinomas, melanomas, lymphomas and sarcomas, some of which are quite often diagnosed in humans. Here, thyroid carcinomas, small-cell lung cancer, gastrointestinal stromal tumours and endometrial carcinomas displayed noticeable C5aR1 expression in a high percentage of samples. Because this may be of therapeutic relevance in the future, further investigations with larger numbers of cases are warranted in these tumour entities.

## Conclusions

With the help of our novel antibody, we were able to detect C5aR1 expression in a wide variety of non-neoplastic and neoplastic tissues. In addition to confirming published findings, we found noticeable C5aR1 expression in many tumour entities, such as in thyroid carcinomas, small-cell lung cancer, gastrointestinal stromal tumours and endometrial carcinomas, for the first time. Here, as well as in other strongly C5aR1-positive tumour entities, C5aR1 may serve as an interesting target for future therapies.

## Supporting information

S1 Raw images (PDF)

## Abbreviations

C5a: complement peptide 5a
C5aR1: complement peptide 5a receptor type 1
HEK: human embryonic kidney
IRS: immunoreactive score
