# Supplementary figures and images for: Immunohistochemical identification of complement peptide C5a receptor 1 (C5aR1) in non-neoplastic and neoplastic human tissues
Source: PLoS One. 2021 Feb 19;16(2):e0246939. doi: 10.1371/journal.pone.0246939 (PMC7894821; doi:10.1371/journal.pone.0246939)

26.7.17

Figure 2A  
left panel

hCSaR1  
5227

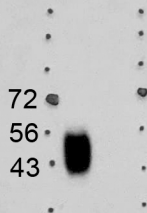

hCSaR1  
hCSaR2

29.6.2018

Figure 2A  
right panel

0.1 µg/µl

CSaR2 1:500 hCSaR1  
5236 HA 5227  
| X X | X X |

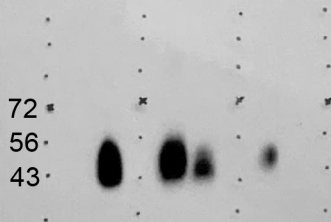

hCSaR1  
hCSaR2

Supplement: S1 Raw images — (PDF) [file pone.0246939.s001.pdf]
